# Beyond the Classroom: The Role of Social Connections and Family in Adolescent Mental Health in the Transylvanian Population of Romania

**DOI:** 10.3390/medicina61061031

**Published:** 2025-06-02

**Authors:** Alexandra-Ioana Roșioară, Bogdana Adriana Năsui, Nina Ciuciuc, Dana Manuela Sîrbu, Daniela Curșeu, Ștefan Cristian Vesa, Codruța Alina Popescu, Monica Popa

**Affiliations:** 1 Department of Community Medicine, Iuliu Hațieganu University of Medicine and Pharmacy, 400349 Cluj-Napoca, Romania; alexandra.rosioara@umfcluj.ro (A.-I.R.); nina.ciuciuc@umfcluj.ro (N.C.); dsirbu@umfcluj.ro (D.M.S.); dcurseu@umfcluj.ro (D.C.); monica.popa@umfcluj.ro (M.P.); 2 Research Center in Preventive Medicine, Health Promotion and Sustainable Development, Iuliu Hațieganu University of Medicine and Pharmacy, 400349 Cluj-Napoca, Romania; 3 Department of Pharmacology, Iuliu Hatieganu University of Medicine and Pharmacy, No. 23 Marinescu Street, 400337 Cluj-Napoca, Romania; stefan.vesa@umfcluj.ro; 4 Department of Abilities Human Sciences, Iuliu Hațieganu University of Medicine and Pharmacy, 400012 Cluj-Napoca, Romania; cpopescu@umfcluj.ro

**Keywords:** adolescent health, schools, risk taking, protective factors, nutritional status, lifestyle, mental health, bullying, sleep–wake disorders, anxiety

## Abstract

*Background and Objectives*: This study explores gender variations in the associations between lifestyle choices, mental health, and social behaviors among adolescents in the Transylvania region of Romania. The analysis is based on data obtained through the Global School-Based Student Health Survey (GSHS). *Materials and Methods*: Data on 900 Romanian adolescents aged 11–18 years were obtained via the GSHS. This study evaluated nutritional statuses through BMI Z-scores, employing World Health Organization (WHO) cut-offs applied to self-reported height and weight; furthermore, it assessed well-being and perceived health; worries and sleep anxiety; social connections through having friends, loneliness, peer support, and emotional support; parental bonding relations; experiences of being bullied; safety and protection factors, including distance learning during the COVID-19 pandemic and testing or vaccinations for COVID-19; and social behaviors, including the use of social networks. A multiple logistic regression was used to predict sleep disturbance anxiety, depending on sex, bullying, cyberbullying, loneliness, social network use, and peer support. *Results*: Results showed that the majority of the adolescents reported having one or more friends (96.8%), with no significant difference between girls and boys (*p* = 0.071). There were no statistically significant differences in bullying and cyberbullying experiences between sexes (*p* = 0.063). Notably, gender disparities exist in both health perceptions and risk behaviors, with girls experiencing higher rates of negative health perceptions, sleep anxiety (*p* < 0.001), and loneliness (*p* = 0.011) and boys exhibiting more overweight/obesity (*p* < 0.001) and school truancy (*p* = 0.027). According to the results, loneliness is significantly associated with a higher likelihood of sleep-disturbing anxiety (*p* < 0.001). Students who have experienced cyberbullying are more likely to also experience traditional bullying. Students who feel lonely are more likely to be victims of bullying. *Conclusions*: This study reveals significant gender disparities in adolescent health, particularly in mental health, risk behaviors, and social support. It highlights the need for gender-specific interventions to address these challenges and promote healthy development. Furthermore, this study emphasizes the importance of social connections, family support, and parental involvement in adolescent well-being. Addressing bullying, promoting mental health awareness, and providing accessible support services are crucial for improving adolescent health in Romania.

## 1. Introduction

The adolescent period, spanning from ages 10 to 19, represents a crucial phase of development characterized by substantial transformations. Despite a common perception of adolescents being healthy, this stage is marked by the emergence of significant health risks that are frequently underestimated [1].

The WHO defines mental health as a state of well-being that equips individuals to manage life’s stresses, realize their potential, facilitate learning and productivity, and contribute to their communities [2]. Considered a fundamental aspect of overall health, it underpins our capacity for decision making, relationship building, and shaping our environment and is recognized as a basic human right, essential for personal, communal, and socio-economic advancement. The WHO also notes that exposure to adverse social, geopolitical, economic, and environmental conditions, such as poverty, inequality, violence, and environmental degradation, elevates the risk of mental health issues [2]. Mental health in adolescence is becoming increasingly important because, globally, mental disorders affect one in seven adolescents aged 10–19 years, contributing to 15% of the overall disease burden in this age group [3]. Depression, anxiety, and behavioral disorders are significant contributors to illness and disability among adolescents, and suicide ranks as the third leading cause of death for individuals aged 15–29 years [3]. Sex-based differences in mental health issues are reported in this age group, with a South Korean study in 2020 showing that females were more depressed, aggressive, and likely to develop somatic symptoms than males, while males exhibited more attention deficits than females [4]. Another study conducted on 2496 adolescents in 2024 in Benin showed that the prevalence of anxiety-induced sleep disturbances was higher among male than female participants [5]. One study analyzing data from 11,440 Chinese adolescents showed that boys reported lower anxiety, were more likely to perpetrate school bullying, and were less likely to engage in dietary restriction compared to girls, while girls in sexual minority groups (specifically bisexual and gay/lesbian) were at a higher risk of eating disorder behaviors [6]. Another study from Spain on 1155 participants addressing bullying and self-concepts shows that, overall, girls show more victimization and boys show more aggression [7].

Social support and self-esteem are associated with a lower likelihood of bullying and cyberbullying victimization, but the exposure to any form of violence increases the risk, even with protective factors [8]. Preventing all forms of violence during childhood and adolescence appears to be the most effective way to protect against bullying and cyberbullying [8]; the other effective ways are education and revising the clinical psychological practices and assessments and the legal policies regarding these topics [9]. Given the interconnected nature of various forms of bullying, a comprehensive, community-wide strategy is essential to effectively address all types of victimization, rather than treating each in isolation. Furthermore, the observed age differences in bullying behaviors suggest that anti-bullying programs should be tailored to specific age groups for maximum effectiveness [10]. Romania is home to over four million children aged 0–18, constituting 21% of the total population of the country [11]. Annually, approximately 9% of this child population requires mental health services [12]. The mental health of Romanian children and teenagers is much more impacted than that of young people from abroad. A concerningly high proportion of Romanian adolescents aged 11–15 (almost 33%) reported experiencing sadness more than once weekly, significantly exceeding the 13% average observed across 45 countries in a WHO study [13]. Further evidence from a 2020 Romanian study involving over 10,000 teenagers (average age: 17 years) revealed that 48.9% of these teenagers had experienced suicidal ideation at least once, 27.1% reported persistent and inescapable sadness, and 21.5% reported intermittent depression in the preceding six months [14]. Romanian adolescents who frequently encounter negative experiences exhibit a heightened susceptibility to depressive and anxiety symptoms [15]. Moreover, the suicide rate of Romanian teens under 15 years of age has increased compared to the average suicide rate of European teens [16]. The most recent national mental health report [17] assesses the prevalence of diagnosed mental disorders in adolescents and identifies conduct disorders (24.19%), attention deficit hyperactivity disorder (ADHD) (22.65%), and anxiety disorders (19.23%) as the most frequently diagnosed mental health conditions [17]. In addition, Romanian children and young people from disadvantaged backgrounds face a heightened vulnerability to mental health issues. For example, over 40% of the newly identified cases of autism spectrum disorders among adolescents originate from rural regions [18] where rehabilitation interventions and therapy are often unavailable. This increased occurrence contrasts sharply with the low proportion of children diagnosed with these disorders and subsequently receiving specialized care [19], a disparity corroborated by expert interviews. Many mental disorders develop early, with 50% emerging before the age of 15 and 75% emerging by adulthood [13]; furthermore, the COVID-19 pandemic has exacerbated mental health challenges for students, as remote learning disrupts essential routines and increases potential dropout rates—even the related evidence from the pandemic is linked more to university students in Romanian studies [20,21]. Social Networking Site (SNS) use is associated with mental health problems in young people (e.g., the potential effects of Snapchat on self-esteem and of TikTok on body image), as are loneliness, Facebook use in 13–15 year olds (linked to decreased family satisfaction, depression, and addiction), and perfectionism (related to internet addiction, risky behaviors, and reduced happiness) [22,23,24]; however, while Romania has low rates of internalizing problems compared to other European countries, adolescent life satisfaction is lower, with high income inequality and negative self-perceptions [25].

There are very few studies in Romania examining the mental health of adolescents, including concepts such as bullying, anxiety-induced sleep disturbances, or peer support in this age group; thus, this study endeavors to provide insights that can help policymakers in promoting mental health awareness and providing accessible support services that are crucial to Romanian society.

This study aims to investigate gender differences in the interrelationships between specific lifestyle choices (nutritional status and sleep hours), mental health (sleep disturbance anxiety), and social behaviors (loneliness, bullying, and peer support) among adolescents in the Transylvania region of Romania.

## 2. Materials and Methods

### 2.1. Population Selection and Study Design

We designed a cross-sectional study that utilized a standardized questionnaire, distributed both online and physically, to investigate mental health and well-being among 900 Romanian middle and high school students aged 11 to 18 years, from Transylvania, during the academic year 2023–2024.

Health behaviors and risk factors in students aged 11 to 18 years were evaluated using the GSHS [26], a standardized survey developed by the World Health Organization (WHO) and the Centers for Disease Control and Prevention (CDC) along with the United Nations International Children’s Emergency Fund (UNICEF), United Nations Educational, Scientific and Cultural Organization (UNESCO), and Joint United Nations Programme on HIV/AIDS (UNAIDS). The GSHS aims to generate information on adolescent protective factors and health behaviors to assist countries in establishing priorities, developing interventions, and advocating for resources that support youth health policies, school health programs, and prevention efforts targeting specific risk factors and vulnerable adolescent groups [26]. While the methodological protocol of this study shares some elements with our previously published work [27], due to the use of the same sample of children, it utilizes a different set of modules from the GSHS questionnaire. Firstly, the research aim is different, focusing on mental health issues and social behaviors of adolescents. Secondly, it uses different variables from those used in our previous work [27], and these variables are explained in Section 2.2 Questionnaire Measurements and Data Collection and include having friends, loneliness, school truancy, peer support, emotional support, parental bonding relations, connectedness, supervision and checks, safety, and protective factors. Thirdly, this study assesses different outcomes, such as bullying experience and anxiety-induced sleep disturbances. Lastly, it uses a subset of data regarding the nutritional status of the adolescents in a novel way, which were used to investigate the association between the anxiety-induced sleep disturbance and the perceived heath status in general.

Romania has a population of 2,132,738 adolescents aged 11 to 18 years [28], of which 72% (*n* = 1,318,298) are enrolled in formal education [29], primarily within the public school sector (97.7%) [30]. This study focuses on this population group. We calculated the representative sample size for our study using Paniott’s formula, with a confidence level of 98% and a margin of error interval of 4% and it was a total of 848 responders. The respondents were selected using convenience samples. We randomly selected schools first and included all students from those selected clusters. We invited schools from counties Cluj, Mures, Alba, Arad, and Nasaud, with a total of 1400 students invited. There were 1120 students who responded and completed the questionnaire, yielding a response rate of 80%. After collecting the questionnaire, it was constated that 120 of the responders did not have the parental consent signed, so they were excluded. After analyzing all the questionnaires, the incomplete questionnaires (*n* = 100) were excluded, leading us to 900 eligible respondents. The inclusion criteria for participants were as follows: a current enrollment in a gymnasium or high school in both rural or urban areas and an age between 11 and 18 years. Exclusion criteria comprised the following: not attending school, being outside the 11–18 age range, a lack of parental consent, and incomplete questionnaires.

**Figure 1:**
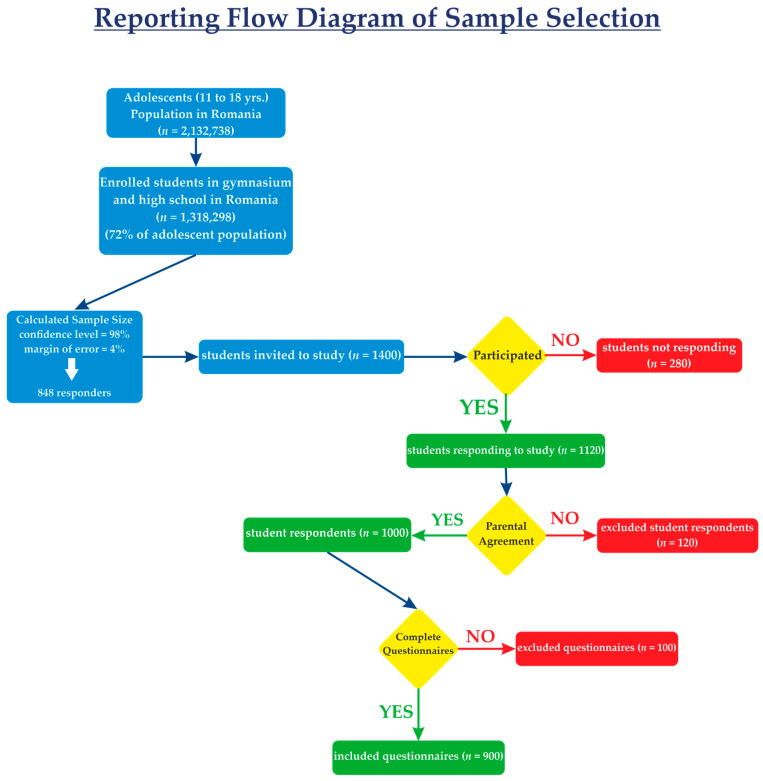
The reporting flow diagram of the sample selection of this study.

Figure 1 illustrates the flow diagram of the study sample, with blue indicating our actions, yellow highlighting data collection milestones, red denoting excluded students and questionnaires, and green representing the included participants.

### 2.2. Questionnaire Measurements and Data Collection

The GSHS core modules evaluate the following ten modules: alcohol use; dietary behaviors; drug use; hygiene; mental health; physical activity; protective factors; sexual behaviors that contribute to HIV infection, other sexually transmitted infections, and unintended pregnancy; tobacco use; violence; and unintentional injury [26]. In this study, the basic core modules were used with selected questions including mental health (4 questions), violence and unintentional injury (6 questions), protective factors (6 questions), dietary behaviors (2 questions), and extended modules including social network use and time spent online (5 questions) and the COVID-19 pandemic (4 questions) in order to assess the factors that influence the mental health and well-being of adolescents [11]. Drawing from the comprehensive GSHS, relevant questions were purposefully selected and grouped into several key domains to align with the study’s specific aims of exploring gender variations in lifestyle choices, mental health, and social behaviors among Romanian adolescents. These domains were designed to capture the multifaceted aspects of adolescent well-being influenced by the COVID-19 pandemic and social contexts. Specifically, “Well-being and Health”: this category encompassed questions designed to assess adolescents’ general health perceptions, nutritional status (derived from self-reported height and weight), and sleep patterns, including anxiety-induced sleep disturbance, which is a key mental health indicator; “Social Connections”: questions in this section evaluated various aspects of adolescents’ social support networks, including the presence of close friends, experiences of loneliness, perceived peer support within the school environment, and the availability of emotional support; “Parental Bonding Relations:” this domain focused on the quality of adolescents’ relationships with their parents or guardians, assessing perceived parental understanding (connectedness), supervision, and active checking-in on their activities; ”Bullying”: this category included questions on both traditional bullying experienced on school property and cyberbullying via social networks, along with inquiries about the source and reasons behind bullying incidents; “Safety and Protection Factors”: this section assessed health-protective behaviors such as seatbelt and helmet use, as well as specific practices related to the COVID-19 pandemic, including mask-wearing, experience with distance learning, and COVID-19 testing or vaccination history; “Social Behaviors—Use of Social Networks”: this category included questions on daily screen time and specific usage patterns of social networks, along with parental rules regarding screen time and mobile phone ownership.

Drawing from the comprehensive GSHS, relevant questions were purposefully selected and grouped into several key domains to align with the study’s specific aims of exploring gender variations in lifestyle choices, mental health, and social behaviors among Romanian adolescents. These domains were designed to capture the multifaceted aspects of adolescent well-being influenced by the COVID-19 pandemic and social contexts, and include:

- Well-being and Health: This category encompassed questions designed to assess adolescents’ general health perceptions, nutritional status (derived from self-reported height and weight), and sleep patterns, including anxiety-induced sleep disturbance, which is a key mental health indicator.
- Social Connections: Questions in this section evaluated various aspects of adolescents’ social support networks, including the presence of close friends, experiences of loneliness, perceived peer support within the school environment, and the availability of emotional support.
- Parental Bonding Relations: This domain focused on the quality of adolescents’ relationships with their parents or guardians, assessing perceived parental understanding (connectedness), supervision, and active checking-in on their activities.
- Bullying: This category included questions on both traditional bullying experienced on school property and cyberbullying via social networks, along with inquiries about the source and reasons behind bullying incidents.
- Safety and Protection Factors: This section assessed health-protective behaviors such as seatbelt and helmet use, as well as specific practices related to the COVID-19 pandemic, including mask-wearing, experience with distance learning, and COVID-19 testing or vaccination history.
- Social Behaviors—Use of Social Networks: This category included questions on daily screen time and specific usage patterns of social networks, along with parental rules regarding screen time and mobile phone ownership.

Nutritional status was evaluated using the Body Mass Index (BMI), calculated from self-reported weight and height data (BMI = Weight [kg]/Height^2^ [m^2^]). The BMI was calculated from self-reported height and weight and then converted to BMI Z-scores based on WHO cut-offs for age and sex. Participants were then classified as underweight, normal weight, overweight, or obese based on the World Health Organization (WHO) cut-off criteria [27,31].

A sample of 900 students from diverse rural and urban schools in the northwest regions of Romania voluntarily and anonymously completed the questionnaire during the 2023–2024 academic year (September 2023–June 2024). Questionnaires that were incomplete or lacked parental consent were excluded from the analysis. After obtaining signed agreements with school administrations, questionnaires were distributed in both online and paper formats to the teachers, to distribute them in class to the responders. The vast majority of the questionnaires were distributed in the paper format, as only one school from one of the included cities decided to use the online format. For the online administration, a parental consent form containing a QR code on the back of the paper was distributed. After the parents signed the informed consent, the adolescents could access the QR code that directed the participants to the questionnaire in order to complete it. For the paper-based questionnaires, students were given the consent form and questionnaire to take home, secure parental consent, and then complete the survey. The estimated time for completion was 20 min. For internal validation, the questionnaire underwent a translation into Romanian and a back-translation into English by a certified translator to ensure the original meaning of the items was preserved. To assess reliability, the instrument was pretested on 30 adolescents in the same age range. Linguistic validation was conducted, and internal consistency was measured on a pilot sample, yielding a Cronbach’s alpha of 0.75 [27]. In the main study sample, internal consistency for the mental health and social support modules yielded Cronbach’s alpha values of 0.76 and 0.71, respectively.

The specific questions and coding schemes for the variables included in the analysis are detailed in Table 1, and coding schemes are performed in accordance with the interpretation guide provided by the GSHS standardized questionnaire [26].

### 2.3. Ethical Considerations

Conducted in accordance with the Declaration of Helsinki guidelines, this study was approved by The Ethics Committee of the Cluj Napoca University of Medicine and Pharmacy (Approval No. 179/20 September 2024). Additionally, we obtained signed agreements from the school administration to facilitate data collection, and all participating adolescents’ parents provided signed informed consent.

### 2.4. Statistical Analyses

All statistical analyses were conducted using IBM SPSS Statistics (version 21, IBM Corp., Armonk, NY, USA) and Microsoft Excel (Microsoft Office 2010, Albuquerque, NM, USA). Descriptive and inferential analyses were performed to address the study’s research questions regarding adolescent health behaviors and their determinants.

Continuous variables (age and BMI) conformed to normality of distribution, verified with the Shapiro–Wilk test; hence they are presented as mean ± standard deviation (SD). All other study variables are categorical and are summarized as frequency (*n*) and percentage (%). Differences between two groups (e.g., boys vs. girls) were assessed with the independent samples *t*-test. Overall associations between categorical variables were examined with the Pearson χ^2^ test. For contingency tables containing more than two column (or row) categories, pair-wise differences were explored post hoc using z-tests for two proportions. A Bonferroni correction was applied to control the family-wise error rate (adjusted *p* = 0.05/number of pair-wise comparisons). Two multivariate logistic regression models were fitted to identify independent predictors of anxiety-induced sleep disturbance and bullying victimization. Independent variables were selected if they achieved statistical significance in univariate analysis. Results are reported as follows: B (log-odds coefficient), adjusted odds ratio (OR) with 95% confidence interval (CI), and *p*-value. Model fit was evaluated with the Hosmer–Lemeshow goodness-of-fit test and Nagelkerke pseudo-R^2^.

A two-sided significance level of *p* < 0.05 was used, except where Bonferroni adjustments were applied.

## 3. Results

### 3.1. Demographic Characteristics of the Study Group

The final sample comprised 900 students from Transylvania, Romania, attending public schools from grades 5 to 12, with ages ranging from 11 to 18 years. The mean age of the participants was 15.5 years (±1.92 SD). Regarding their residence, more than half of the responders were from urban areas, and the rest of them were from rural areas. The gender distribution of the sample was 52.7% female and 47.3% male. Table 2 provides an overview of the demographic characteristics of the participants, including details on their age, sex, class, and residential area.

### 3.2. Health Perceptions and Well-Being

This study conducted a descriptive analysis regarding well-being and health perceptions. As shown in Table 3, 86.4% of all responders had a positive perception about their health, meaning that they perceived their health as good or very good [32]. A significantly larger percentage of girls reported a negative perception of their health status compared to boys (*p* < 0.001). Conversely, a significantly higher proportion of boys were classified as overweight or obese (*p* < 0.001). No significant gender difference was observed in the average number of sleep hours per night (*p* = 0.118). However, girls reported experiencing sleep anxiety significantly more often than boys (*p* < 0.001).

### 3.3. Social Connections and Peer Support

This study examined the various aspects of social connections and peer support among Romanian adolescents, with a breakdown by gender, as seen in Table 4.

The vast majority of adolescents report having friends (96.8%), with no significant difference between girls and boys, as a slightly higher percentage of girls reported having friends compared to boys (*p* = 0.071). Regarding loneliness, more girls report feeling lonely compared to boys (*p* = 0.011). The higher prevalence of loneliness among girls raises concerns and warrants further investigation into the underlying causes and potential interventions. When talking about school truancy, boys are more likely to report skipping school than girls (*p* = 0.027). Boys are more likely to report having kind and helpful colleagues/peers compared to girls (*p* = 0.016). The association between school truancy and lower levels of peer support, particularly among boys, suggests that addressing truancy could involve strengthening peer relationships and creating a more supportive school environment. Regarding having emotional support and having someone to discuss their problems with, there were no significant differences between girls and boys in terms of receiving emotional support (*p* = 0.076). While there was no significant gender difference in receiving emotional support, it is crucial to consider the quality and source of this support, as well as the potential unmet needs.

### 3.4. Parental Bonding Relations

This study also explored the relationship between Romanian adolescents and their parents or guardians, focusing on supervision, connectedness, and checking-in, as showed in Table 5. When talking about the connection they have with their parents, more boys than girls report feeling connected to their parents or guardians (*p* = 0.043). Also, more boys than girls report having parental or guardian supervision (*p* = 0.03). The data suggest that boys feel more connected to and supervised by their parents or guardians compared to girls. This could reflect differences in parenting styles or expectations for boys and girls. It is important to explore the reasons behind this disparity and its potential implications for adolescent well-being. Regarding homework check-ups, there is no significant difference between girls and boys in whether their parents or guardians check on them (*p* = 0.873). While there is no gender difference in parental checking-in, a total of 77% of the responders said they have parental check-ups, and it is important to understand how this behavior contributes to adolescent development and whether it varies across different family structures or cultural contexts.

### 3.5. Bullying

This study also analyzed data on bullying and cyberbullying experiences among responders, including the source and the reason of bullying at school, as seen in Table 6.

There were no statistically significant differences in bullying experiences between sexes (*p* = 0.063). Similarly, there was no significant difference in cyberbullying experiences between girls and boys (*p* = 0.233). Only 10.4% of the responders initially said they have been bullied, and 8.6% said that they have been cyberbullied; after those two questions, when they were asked about the source of bullying with the question “who was the person that bullied you most often?”, where the answers were “students in my school/students from another school/another person my age”, almost half of the responders admitted that they have been bullied. The most common reason for bullying is related to physical appearance and ethnicity (10.9%), followed by religion and personal beliefs (2.0%), sexual orientation and gender (1.4%), and income or social status (0.9%). There is no significant difference between girls and boys in terms of the reason for the bullying (*p* = 0.22). The data reveal that physical appearance and ethnicity are the most common reasons for bullying, underscoring the importance of addressing prejudice and promoting inclusivity in schools and communities. With the Bonferroni correction for the five bullying reason categories the difference was not statistically significant.

### 3.6. Safety and Protection Factors

Our study also investigated various safety and protection behaviors, as illustrated in Table 7. Regarding the habit of using a seat belt when they were in a car, almost 80% of students responded that they used seat belts, and we found no significant difference between girls and boys in seat belt usage (*p* = 0.68).

More boys wore helmets while bicycling compared to girls (*p* = 0.027), although 93.3% of the responders did not wear helmets while bicycling.

More girls wore COVID-19 masks compared to boys (*p* < 0.001). The data suggest that boys are more likely to wear helmets while bicycling, while girls are more likely to wear COVID-19 masks. This could reflect differences in risk perception, social norms, or parental expectations for boys and girls.

Regarding the use of technology for home-schooling during the COVID-19 pandemic, no significant gender difference was observed (*p* = 1), likely because the vast majority of both girls and boys reported learning via computers or smartphones. This widespread reliance on technology for remote education during the pandemic warrants a further investigation into its long-term effects on learning, social development, and digital equity. Similarly, no significant gender differences were found in the COVID-19 testing history (*p* = 0.08) or vaccination status (*p* = 0.955).

### 3.7. Factors Associated with Anxiety-Induced Sleep Disturbance

**Figure 2:**
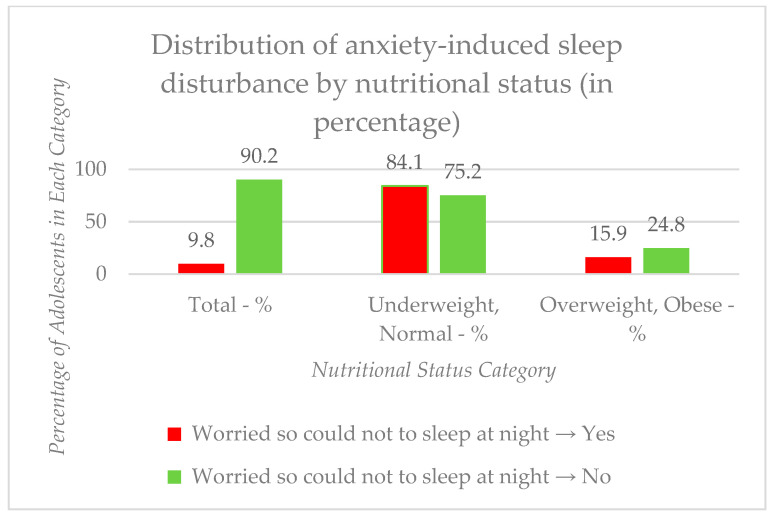
Distribution of anxiety-induced sleep disturbance by nutritional status: categories of underweight and normal weight adolescences and overweight and obese adolescents are expressed in percentages.

To analyze the relationship between “worried so could not sleep at night” (anxiety-induced sleep disturbance) and nutritional statuses, categorized as “under/normal weight” and “overweight/obese”, we used the chi-square test that resulted in a *p*-value of 0.086, which indicates that there is no statistically significant association between experiencing worry that prevents sleep and being in either the “under/normal weight” or “overweight/obese” category. Figure 2 presents the distribution of anxiety-induced sleep disturbances by the nutritional status: the category of underweight and normal weight adolescences and the category of overweight and obese adolescents are expressed in percentages.

Figure 2 presents the distribution of anxiety-induced sleep disturbances across nutritional status categories. Although there was a trend for individuals with a normal weight to report less sleep-disturbing worry, this association did not reach statistical significance (χ^2^ = 3.416^a^, df = 1, *p* = 0.086).

**Figure 3:**
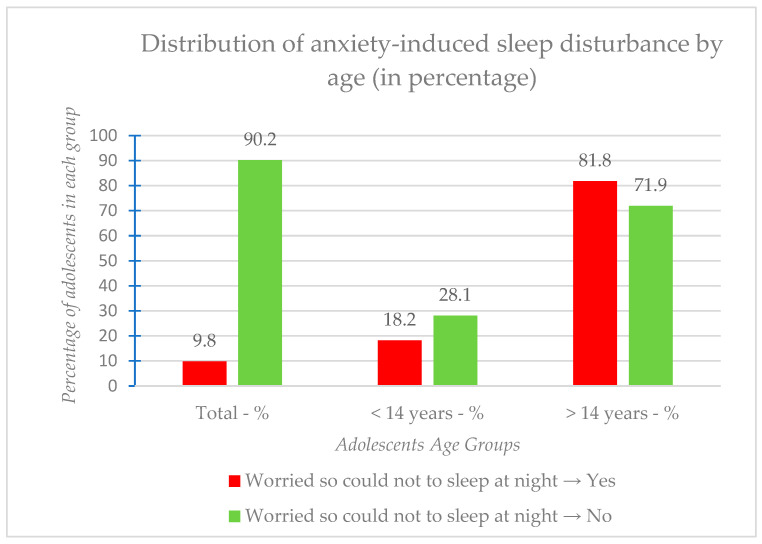
Distribution of anxiety-induced sleep disturbance by age range.

Moreover, the relationship between age and anxiety-induced sleep disturbance was explored using the chi-square test, resulting in a *p*-value of 0.063. This shows that the association between the worry that prevents sleep and age group is not statistically significant. Figure 3 presents the distribution of anxiety-induced sleep disturbances by age groups in percentages.

As illustrated in Figure 3, while a higher percentage of adolescents over 14 years reported sleep-disturbing worry compared to those under 14, this difference was not statistically significant (χ^2^ = 3.935^a^, df = 1, *p* = 0.063).

A multiple regression model was used to predict the anxiety-induced sleep disturbance, depending on sex, bullying, cyberbullying, loneliness, social network use, and peer support, as seen in Table 8. According to the results, loneliness is significantly associated with a higher likelihood of sleep-disturbing anxiety (*p* < 0.001). Being a girl (compared to a boy) is associated with a significantly lower likelihood of experiencing sleep-disturbing anxiety (*p* < 0.001). Having emotional support is associated with a lower likelihood of sleep-disturbing anxiety (*p* = 0.011). Feeling connected to parents or guardians is associated with a lower likelihood of sleep-disturbing anxiety (*p* = 0.008). The logistic model explained 27.7% of the variance in the outcome (Nagelkerke R^2^ = 0.277), which indicates a moderate explanatory power. The model showed an adequate fit to the data (Hosmer–Lemeshow chi-square (7) = 1.082, *p* = 0.9).

### 3.8. Exploring the Relation Between Bullying and Contributing Factors

A multiple regression was performed to predict the experience of being bullied based on cyberbullying, having or not having friends, being able to talk about problems with someone, being able to talk about problems with parents, feeling lonely, social support (kind students), and sleep (hours/night), as seen in Table 9. The results show that the positive coefficient (1.927) and statistical significance (*p* ≤ 0.001) indicate that students who have experienced cyberbullying are more likely to also experience traditional bullying. The negative coefficient (−0.714) and lack of statistical significance (*p* = 0.098) suggests that students who have friends are less likely to be victims of bullying. The negative but statistically insignificant coefficients indicate that talking about problems with someone or with parents does not have a significant influence on the probability of experiencing bullying, according to this model. Students who feel lonely are more likely to be victims of bullying (*p* ≤ 0.001). The logistic model explained 13.1% of the variance in the outcome (Nagelkerke R^2^ = 0.131), which indicates a moderate explanatory power. The model showed an adequate fit to the data (Hosmer–Lemeshow chi-square (7) = 11.93, *p* = 0.1).

A second model of regression was generated to predict the factors that can influence bullying (using the other variable—source of bullying—in the last year who bullied you the most (Yes/No)) and included variables like age, cyberbullying, loneliness, the use of social networks, and having peer support, as presented in Table 10. Variables that achieved significance were age, cyberbullying, and peer support. The logistic model explained 13.1% of the variance in the outcome (Nagelkerke R^2^ = 0.181), which indicates a moderate explanatory power. The model showed an adequate fit to the data (Hosmer–Lemeshow chi-square (8) = 4.37, *p* = 0.8).

## 4. Discussion

This study’s aim was to investigate gender differences in the interrelationships between specific lifestyle choices (nutritional status, sleep hours, and protective and risk factors), mental health (sleep disturbance and anxiety), and social behaviors (loneliness, bullying, and peer support) among adolescents in the Transylvania region of Romania. Regarding overall health and well-being, the findings of this study underscore statistically significant differences between male and female participants in several key aspects. Specifically, girls reported negative perceptions of their health more often (*p* < 0.001). This result is in line with those of other international studies that explored sex differences in health perceptions at different ages [33,34,35]. In addition, another finding is that anxiety-induced sleep disturbance was more frequent in girl responders (*p* < 0.001). This finding is congruent with those of a study of 3778 young Australians [36], which indicated a higher prevalence of poor sleep quality in females than males, and a Benin study on the same topic with 2694 adolescents [5], which indicated girls are more worried than boys and do not sleep well at night. Regarding friends and loneliness, our results show that although more than 96.8% of the responders said that they have friends, a slightly higher percentage of girls reported having friends compared to boys (*p* = 0.046); although 85.4% of all responders said that they do not feel lonely, girls are more likely to feel lonely than boys (*p* = 0.011). These results are consistent with those of a 2024 meta-analysis that examined gender differences in well-being [37]. In contrast, the results of this study showed that boys had a higher prevalence of overweight/obesity (*p* <0.001) and risk behaviors such as school truancy (*p* = 0.027), which is similar to findings of another study using the same methodology as this study and conducted in the United States of America (USA) on a sample of 9016 adolescents regarding nutritional status [38], as well as other behaviors, such as school absenteeism; a study in the USA on 23,459 adolescents of the same age range [39]; a study from Benin using the same methodology [40]; and a meta-analysis regarding the risk factors for school absenteeism [41]. Overall, 59.1% of all the responders sleep less than 8 h per night, which is recommended [42,43], with no statistically significant differences found between sexes. These differences highlight the need for gender-specific interventions and support systems.

The study results evidenced that boys are more likely to report having kind and helpful colleagues/peers compared to girls (*p* = 0.016), and this result contrasts those of other studies from abroad [44,45]. Regarding emotional support from family or friends, 58.6% of the responders said they do not have anyone to talk to about their problems, with no statistically significant differences between sexes (*p* = 0.076); in contrast, in a recent report from the USA regarding the perceived social and emotional support among teenagers in 2022, 58.5% of the teenagers reported that they received adequate emotional support [46]. Strong social connections are associated with better mental health, reduced risk behaviors, and increased resilience in adolescents [47], aligning with the growing recognition of the importance of support from peers, friends, and family, which is supported by other international studies [48,49]. Therefore, interventions should focus on strengthening these relationships.

This study investigated parental bonding relations, and 62.2% of the responders said that they have parental connectedness; more boys than girls reported feeling connected to their parents or guardians (*p* = 0.043). Moreover, more boys than girls reported having parental or guardian supervision (*p* = 0.03). Regarding parental checks, there was no significant difference between the sexes, and 77.1% of all responders said they have regular parental checks. Parental influence through parental connection, supervision, and communication is an important factor in adolescent development, and our results are consistent with those of other international studies that support our findings [48,49,50,51]. The data from this study suggest the potential gender differences in these relationships, warranting further investigation and tailored interventions to support healthy family dynamics.

Bullying and cyberbullying remain significant concerns [8,9,10,52], with potential long-term consequences for victims. Although the study results initially showed that 10.4% of surveyed adolescents admitted to having experienced bullying, when asked who had bullied them in the past 12 months, whether the source was other peers or acquaintances at their school or another school, 48.8% of responders mentioned one of the aforementioned sources of bullying. A comprehensive approach involving schools, families, and communities is needed to prevent bullying and provide support to those affected.

This study’s cross-sectional design inherently presents several limitations. Firstly, while associations between factors and outcomes were observed, causality cannot be definitively established. Secondly, the reliance on self-reported data regarding attitudes and behaviors introduces the potential for social desirability bias, possibly leading to an overstatement of positive health behaviors and an understatement of less favorable ones, such as smoking and alcohol use; also, self-reported data regarding height and weight can represent a potential inaccuracy and bias at this age of adolescence. Thirdly, the accuracy of adolescents’ self-reported behaviors and health information may have been affected by recall bias. Fourthly, this study’s geographical scope, limited to the Transylvanian region, restricts the generalizability of the findings to all Romanian adolescents. Fifthly, some of the relevant confounders, like the family structure and pre-existing health conditions, were not explored in this study, which could influence the observed associations. Lastly, the data collection within the school setting might have introduced a peer influence on the responses.

Despite these acknowledged limitations, this study’s high response rate and geographically varied sample offer robust evidence regarding the influence of modifiable environmental factors—including parental rules, school policies, and community resources—on adolescent health, providing valuable insights for promoting lifelong well-being.

Future research should use longitudinal designs for the following: to examine the long-term effects of factors like bullying and anxiety-induced sleep disturbances on adolescent mental health; to investigate how these relationships evolve over time, particularly during critical developmental periods; and to assess the directionality of effects (e.g., does loneliness lead to increased social media use or vice versa?). Moreover, our study identifies risks and protective factors. We suggest that future research should use this information to develop and evaluate interventions for the following: to enhance social support and reduce loneliness among adolescents; to develop and test intervention programs aimed at reducing bullying and promoting positive online interactions; and to evaluate the effectiveness of interventions targeting sleep disturbance anxiety in adolescents.

## 5. Conclusions

This study underscores the complex interplay of gender, social factors, and mental health among Romanian adolescents from Transylvania. Notably, gender disparities exist in both health perceptions and risk behaviors, with girls experiencing higher rates of negative health perceptions, sleep anxiety, and loneliness and boys exhibiting a higher rate of overweight/obesity and school truancy. Furthermore, strong social connections and parental involvement emerge as protective factors against sleep-disturbing anxiety, while loneliness and cyberbullying significantly increase the risk of bullying. These findings highlight the urgent need for gender-sensitive interventions and comprehensive strategies that strengthen social support systems, promote mental health awareness, and address bullying to foster healthy adolescent development in Romania.

This study provides valuable insights into the health and well-being of Transylvanian/Romanian adolescents, highlighting the need for comprehensive and gender-specific interventions to promote healthy development and address the challenges faced by young people in today’s society.

## Figures and Tables

**Table 1:** Questions and coding schemes for the variables included in the analysis.

Variables	Questions	Coding Schemes
**Socio-demographic**		
Age [27]	How old are you?	11 or younger, 12–14 “≤14” 15–18 or older “≥15” [27]
Sex	What is your sex?	Female “Girls”; Male “Boys” [27]
Background	What residential area do you live in?	“Rural”; “Urban”
**Well-being and Health**		
Nutritional status (calculated using BMI WHO Z-score cut-off [31])	“How tall are you without shoes?“ [26] “What is your weight?” [26]	<−2 SD from median for BMI by age and sex “Underweight” >+1 SD from median for BMI by age and sex “Overweight” >+2 SD from median for BMI by age and sex “Obese” [31]
Self-perceived health status	“How would you describe your health in general?” [26]	Excellent/very good/good “Positive Perception About Health” [27] Acceptable/poor “Negative Perception About Health”
Worries and sleep anxiety	“In the past 6 months, how often were you so worried about something that you couldn’t sleep at night?” [26]	Most of the time/always “Yes” Never/rarely/sometimes “No”
Sleeping hours per night	“During school time, how many hours do you sleep each night?” [26]	<8 h per night “Not Enough Sleep” ≥8 h per night “Enough Sleep”
**Social connections**		
Having friends	“How many close friends do you have?”(friends you can confine to, you feel safe with) [26]	0 friends “ No—not having fiends” 1 friend/2 friends/3 or more friends “Yes—having friends”
Loneliness	“In the past 6 months, how often have you felt lonely?” [26]	Most of the time/always “Yes” Never/rarely/sometimes “No”
School truancy	“In the past 30 days, how many days were you absent from school without permission? (i.e., you skipped school)” [26]	3 to 5 days/6 to 9 days/10 or more days “Yes” 0 days/1 or 2 days “No”
Kind and helpful colleague/peer support	“In the past 30 days, how often has it happened that most of the students in your school were kind and helpful?” [26]	Most of the time/always “Yes” Never/rarely/sometimes “No”
Emotional support	“In the past 30 days, how often were you able to talk to someone about your problems and worries?” [26]	Most of the time/always “Yes” Never/rarely/sometimes “No”
**Parental bonding relations**		
Parental or guardian connectedness	“In the past 30 days, how often did your parents or guardians understand your problems and worries?” [26]	Most of the time/always “Yes” Never/rarely/sometimes “No”
Parental or guardian supervision	“In the past 30 days, how often did your parents or guardians check on you to see if you did your homework?” [26]	Most of the time/always “Yes” Never/rarely/sometimes “No”
Parental or guardian check	“In the past 30 days, how often did your parents or guardians really know what you were doing in your free time?” [26]	Most of the time/always “Yes” Never/rarely/sometimes “No”
**Bullying**		
Bullying experience	“In the past 12 months, have you been bullied or harassed on school property (by other children or classmates)?” [26]	“Yes” “No”
Cyberbullying experience	“In the past 12 months, have you been cyberbullied (on social networks)?” [26]	“Yes” “No”
Source of Bullying	“In the past 12 months, who bullied you most often?” [26]	No one “No” Students in my school/students from another school/another person my age “Yes”
Reason of Bullying	“In the past 12 months, what was the main reason you were bullied?” [26]	I have not been harassed in the past 12 months “No” Because of the way my body or face looks/because of my disabilities/because of my ethnicity or skin color “Physical and Ethnicity reasons” Because of my gender, sexual orientation, or gender identity “Sexual orientation and gender identity” Because of my religion/because I was good at school “Religion and personal beliefs” Because of how rich or poor my family is “Family income or social status reasons” Other reasons “Others”
**Safety and Protection Factors**		
Seat belt	“In the past 30 days, how often did you wear a seat belt when you were in a car or other motorized vehicle driven by someone else?” [26]	Most of the time/always “Yes” Never/rarely/sometimes “No”
Helmet	“In the last 30 days, how often did you wear a helmet when riding a bicycle?” [26]	Most of the time/always “Yes” I haven’t ridden a bike in the last 30 days/never/rarely/sometimes “No”
COVID-19 Mask	“During the COVID-19 pandemic, how often did you wear a mask or other face covering to protect yourself or others from the disease when in public?” [26]	Most of the time/always “Yes” Never/rarely/sometimes “No”
Home-Schooling with technology (during COVID-19)	“During the COVID-19 pandemic, did you attend school from home at least part of the time using a computer, cell phone, or other electronic devices?” [26]	“Yes” “No”
Testing COVID-19 history	“During the COVID-19 pandemic, were you tested by a doctor or nurse for COVID-19 infection?” [26]	“Yes” “No” “I don’t know”
COVID-19 vaccination	“Have you been vaccinated to prevent infection with COVID-19?” [26]	“Yes” “No” “I don’t know”
**Social behaviors—the use of social networks**		
Screen time (hours/day)	“On a typical school day, how many hours of screen time do you spend?” [26]	</= than 2 h/day: “No risk factor” > than 2 h/day: “Risk factor”
Use of social network	“In the past 7 days, how many hours a day did you use your mobile phone for social networks, for online communication or to surf the Internet?” [26]	</= than 2 h/day: “No risk factor” > than 2 h/day: “ Risk factor”
Parental rules for screen time and social network	“Do your parents or guardians have rules about how you can use social media, online communication or the Internet?” [26]	“Yes” “No”
Having a personal mobile phone	“Do you have your own mobile phone to use?” [26]	“Yes” “No”

**Table 2:** The socio-demographic description of the sample by numbers and percentages.

Variables	Category	N = 900	Percentage (%)
Sex	Girls	474	52.7
	Boys	426	47.3
Age	Under 14	244	27.1
	Over 14	656	72.9
Class	5–8 (gymnasium)	247	27.4
	9–12 (high school)	653	72.6
Residence	Urban	639	71
	Rural	261	29

**Table 3:** Well-being and health perceptions—distribution between boys and girls.

Variable	Item	Total, *n* (%)	Girls, *n* (%)	Boys, *n* (%)	*p*-Value
**Well-Being and Health**					
Perceived health status [27]	Negative perception	122 (13.6)	89 (18.8)	33 (7.7)	<0.001 *
	Positive perception	778 (86.4)	385 (81.2)	393 (92.3)	
Nutritional status [27]	Underweight, normal weight	685 (76.1)	396 (−83.5)	289 (−67.8)	<0.001 *
	overweight, obese	215 (23.9)	78 (−16.5)	137 (−32.2)	
Sleep hours per night	<8	532 (59.1)	292 (61.6)	240 (56.3)	0.118
	≥8	368 (40.9)	182 (38.4)	186 (43.7)	
Worries and sleep anxiety	No	812 (90.2)	407 (85.9)	405 (95.1)	<0.001 *
	Yes	88 (9.8)	67 (14.1)	21 (4.9)	

* *p* < 0.05 was considered statistically significant; chi-square.

**Table 4:** Social connection and peer support—distribution between boys and girls.

Variable	Item	Total, *n* (%)	Girls, *n* (%)	Boys, *n* (%)	*p*-Value
**Social Connections**					
Having friends	No	29 (3.2)	10 (2.1)	19 (4.5)	0.071
	Yes	871 (96.8)	464 (97.7)	407 (95.5)	
Loneliness	No	769 (85.4)	391 (82.5)	378 (88.7)	0.011 *
	Yes	131 (14.6)	83 (17.5)	48 (11.3)	
School truancy	No	844 (93.8)	453 (95.6)	391 (91.8)	0.027 *
	Yes	56 (6.2)	21 (4.4)	35 (8.2)	
Kind and helpful colleagues/peer support	No	512 (56.9)	288 (60.8)	224 (52.6)	0.016 *
	Yes	388 (43.1)	186 (39.2)	202 (47.4)	
Emotional support	No	528 (58.6)	265 (55.9)	263 (61.7)	0.076
	Yes	372 (41.3)	209 (44.1)	163 (38.3)	

* *p* < 0.05 was considered statistically significant; chi-square.

**Table 5:** Parental bonding relations—distribution between boys and girls.

Variable	Item	Total, *n* (%)	Girls, *n* (%)	Boys, *n* (%)	*p*-Value
**Parental Bonding Relations**					
Parental or guardian connectedness	No	340 (37.8)	192 (40.5)	148 (34.7)	0.043 *
	Yes	560 (62.2)	282 (59.5)	278 (65.3)	
Parental or guardian supervision	No	665 (73.9)	365 (77)	300 (70.4)	0.03 *
	Yes	235 (26.1)	109 (23)	126 (29.6)	
Parental or guardian check	No	206 (22.9)	110 (23.2)	96 (22.5)	0.873
	Yes	694 (77.1)	364 (76.8)	330 (77.5)	

* *p* < 0.05 was considered statistically significant; chi-square.

**Table 6:** Bullying experience—distribution between boys and girls.

Variable	Item	Total, *n* (%)	Girls, *n* (%)	Boys, *n* (%)	*p*-Value
**Bullying**					
Bullying experience (ever)	No	806 (89.6)	432 (91.1)	374 (87.8)	0.063
	Yes	94 (10.4)	42 (8.9)	52 (12.2)	
Cyberbullying experience	No	823 (91.4)	437 (92.2)	386 (90.6)	0.233
	Yes	77 (8.6)	37 (7.8)	40 (9.4)	
Source of bullying (last 12 months)	No bullying	461 (51.2)	245 (51.7)	216 (50.7)	0.41
	Yes (colleagues, mates)	439 (48.8)	229 (48.3)	210 (49.3)	
Reason of bullying	No bullying	696 (77.3)	359 (75.7)	337 (79.1)	0.22
	Physical and ethnicity reasons	98 (10.9)	61 (12.9)	37 (8.7)	
	Religion and personal beliefs	18 (2.0)	11 (2.3)	7 (1.6)	
	Sexual orientation and gender identity	13 (1.4)	7 (1.5)	6 (1.4)	
	Family income or social status reasons	8 (0.9)	2 (0.4)	6 (1.4)	
	Other reasons	67 (7.4)	34 (7.2)	33 (7.7)	

Pair-wise z-tests with Bonferroni adjustment were used when χ^2^ revealed overall significance.

**Table 7:** Safety and protection factors—distribution between boys and girls.

Safety and Protection Factors					
Seat belt	No	188 (20.9)	96 (20.3)	92 (21.6)	0.68
	Yes	712 (79.1)	378 (79.7)	334 (78.4)	
Helmet (during bicycling)	No	844 (93.8)	453 (95.6)	391 (91.8)	0.027 *
	Yes	56 (6.2)	21 (4.4)	35 (8.2)	
COVID-19 mask	No	122 (13.6)	41 (8.6)	81 (19)	<0.001 *
	Yes	778 (86.9)	433 (91.4)	345 (81)	
Home-schooling with technology (during COVID-19)	No	13 (1.4)	7 (1.5)	6 (1.4)	1
	Yes	887 (98.6)	467 (98.5)	420 (98.6)	
COVID-19 testing history	No	295 (32.8)	153 (17)	142 (15.8)	0.08
	Yes	538 (59.8)	277 (30.8)	261 (29)	
	I don’t know	67 (7.4)	44 (4.9)	23 (2.6)	
COVID-19 vaccination	No	654 (72.7)	346 (38.4)	308 (34.2)	0.955
	Yes	226 (25.1)	118 (13.1)	108 (12)	
	I don’t know	20 (2.2)	10 (1.1)	10 (1.1)	

* *p* < 0.05 was considered statistically significant; chi-square.

**Table 8:** Multiple regression predicts association between anxiety and factors involved in sleep disorders.

Model	B	*p*	OR	95% CI	
				Min	Max
Sex (male)	−1.109	<0.001 *	0.33	0.191	0.572
Bullying (yes)	0.27	0.475	1.31	0.625	2.745
Cyberbullying (yes)	0.654	0.085	1.924	0.913	4.054
Loneliness (yes)	1.368	<0.001 *	3.928	2.334	6.612
Emotional support (yes)	−0.872	0.011 *	0.418	0.213	0.819
Parental or guardian connectedness (yes)	−0.743	0.008 *	0.476	0.274	0.825
Peer support (yes)	−0.345	0.208	0.708	0.414	1.212
Sleep hours per night (yes)	−0.911	0.004 *	0.402	0.218	0.742
Constant	−2.153	<0.001	0.116		

* *p* < 0.05 was considered statistically significant; CI—confidence interval; OR—Odds Ratio; and B—Unstandardized Regression Coefficient.

**Table 9:** Multiple regression on bullying experience and factors involved in it.

Model	B	*p*	OR	95% CI	
				Min	Max
Cyberbullying (yes)	1.927	<0.001 *	6.868	3.445	13.692
Friends (yes)	−0.714	0.098	0.49	0.21	1.14
Talking about problems with someone (yes)	−0.171	0.264	0.843	0.625	1.138
Talking about problems with parents (yes)	−0.296	0.064	0.744	0.544	1.017
Loneliness (yes)	0.776	<0.001 *	2.173	1.413	3.342
Peer support (yes)	−0.168	0.243	0.846	0.638	1.121
Sleep hours per night (yes)	−0.26	0.076	0.771	0.578	1.028
Constant	1.744	<0.001	5.719		

* *p* < 0.05 was considered statistically significant; CI—confidence interval, OR—Odds Ratio; and B—Unstandardized Regression Coefficient.

**Table 10:** Multiple regression on source of bullying and factors involved in it.

Model	B	*p*	OR	95% CI for OR	
				Min	Max
Age	−0.725	0.004 *	0.484	0.295	0.794
Cyberbullying (yes)	2.03	<0.001 *	7.615	4.397	13.186
Loneliness (yes)	0.483	0.096	1.621	0.917	2.864
Social networks (yes)	−0.456	0.071	0.634	0.387	1.039
Peer support (yes)	−0.813	0.002 *	0.443	0.266	0.739
Constant	0.058	0.892	1.059		

* *p* < 0.05 was considered statistically significant; CI—confidence interval, OR—Odds Ratio; and B—Unstandardized Regression Coefficient.

## Data Availability

The datasets generated and analyzed during this study are not publicly available since they were specifically collected by the authors for this study, but they may be made available by the corresponding author on reasonable request.

## Abbreviations

The following abbreviations are used in this manuscript:

WHO	World Health Organization
GSHS	Global School-Based Student Health Survey
COVID-19	Coronavirus Disease-19
SNS	Social Networking Site
CDC	Centers for Disease Control and Prevention
UNAIDS	Joint United Nations Programme on HIV/AIDS
UNESCO	United Nations Educational, Scientific and Cultural Organization
UNICEF	United Nations International Children’s Emergency Fund
